# Divergent Role of AI in Social Development: A Comparative Study of Teachers’ and Students’ Perceptions in Online and Physical Classrooms

**DOI:** 10.3390/bs15121649

**Published:** 2025-11-30

**Authors:** Qianye Wen, Jianliang Wang, Zhuoqi Guo, Daniel Badulescu

**Affiliations:** 1 School of Education, Central China Normal University, 152 Luoyu Road, Wuhan 430079, China; wangjl@mail.ccnu.edu.cn; 2 Department of Chinese Language and Literature, East China Normal University, 3663 North Zhongshan Road, Shanghai 200062, China; zhuoqiguo77@gmail.com; 3 Department of Economics and Business, University of Oradea, 410080 Oradea, Romania; dbadulescu@uoradea.ro

**Keywords:** AI, sustainable education, social development, academia, technology, online teaching, in-person teaching

## Abstract

This study addresses a critical gap in understanding Artificial Intelligence (AI)’s role in education by empirically investigating and comparing the distinct perceptions of teachers and students regarding AI’s role in a comprehensive range of social development aspects in both online and physical classroom settings. In particular, we evaluated how teachers utilize AI in their teaching methods, namely, Communicative Language Teaching (CLT), the Direct Method (DL), Task-Based Language Teaching (TBLT), Content and Language Integrated Learning (CLIL), and Community Language Learning (CLL), and students in their learning methods, namely, Communicative Learning (CL), Immersive Learning (IL), Task-Based Collaborative Learning (TBCL), Content Integrated Learning (CIL), and Community-Based Reflective Learning (CBRL), to configure their social development. We interviewed 20 teachers (10 from online and 10 from physical classes) and 40 students (20 from online and 20 from physical classes) and evaluated their perceptions regarding AI usage in teaching and learning methods towards social development. The results of our study are convincing enough to suggest that both teachers and students perceive AI usage helpful in teaching models; however, variation in their perception is observed. Notably, the divergence in the perception of teachers and students with regard to AI’s role is a key observation of this study. For instance, the teachers perceived AI as a highly effective tool in fostering community building during online sessions; in contrast, the students viewed its role as being moderately effective. Likewise, the teachers perceived AI’s role as a critical tool in traditional classrooms rather than in virtual ones, whereas the students associated AI with online learning—in terms of digital tools, learning opportunities, and critical discussion—by rating its impact on social confidence and verbal–nonverbal communications significantly more strongly in physical settings. On the contrary, the teachers emphasized AI’s relevance to their self-confidence, emotional intelligence, and community engagement in online teaching platforms; yet, the ratings dropped to moderate in physical contexts. The students’ perceptions in this regard matched those of the teachers, as they also emphasized the importance of social confidence and overall well-being in physical classrooms, where the teachers’ assessment was comparatively low. These patterns provide analytical insights that are decisively valuable for designing AI-integrated pedagogical models that support social development within the educational environments.

## 1. Introduction

Since its advent, Artificial Intelligence (AI) has significantly transformed human life, including our daily activities (Aldoseri et al., 2024; Lee et al., 2023), social engagement (Bokhari & Myeong, 2022), business operations (Anwar, 2024), learning processes (Gligorea et al., 2023), and teaching methods (Salas-Pilco et al., 2022). Given its critical role, participants in academia, including both teachers and students, have analyzed the impact of AI, particularly Generative AI, on individuals’ growth, performance, and social development (Aguilar-Esteva et al., 2023; Mhlanga, 2021; Vieriu & Petrea, 2025). Numerous scholars have stressed the need to investigate this transformative shift to understand the multifaceted influence of AI on educational systems, particularly its role in social development within the educational context (Altinay et al., 2024; Gupta et al., 2024; Lai et al., 2023). Henceforth, it is fundamentally important that we analyze the role of Generative AI and its impact on social skills, emotional intelligence, and collaborative competencies. This provides a deep insight into how AI might affect the learning environment of teachers and students in both traditional classrooms and digital learning platforms.

In the available literature, the majority of studies have encompassed the importance of digital adoption in academia and analyzed its impact on learning outcomes (Sailer et al., 2021; Wu & Yu, 2024; Yeung et al., 2021); yet, its impact on the social development of learners and educators remains underexplored. In the context of our study, social development is defined as the process of interactions (through physical or online classes) between teachers and students that aim to create a productive environment through ideas and valuable knowledge, which is essential for enhancing their social skills, emotional regulations, attitudes, values, and social cognition (Bandura & Walters, 1977; Vygotsky, 1978). While traditional classes involve the in-person interaction of both teachers and students in a physical environment, online learning is reported to be a popular alternative teaching method in which teachers and students interact through a digital platform (such as Zoom, Teams, etc.) (Yu & Jee, 2020). Despite its enrichment, the current literature has completely overlooked the distinct perceptions of both teachers and students regarding Gen-AI’s pivotal role in various social development aspects within online and physical learning environments. Therefore, our study intends to address this significant gap by analyzing how these stakeholders experience and evaluate Gen-AI’s roles. Specifically, this study intends to analyze its role in fostering individuals’ critical social skills, emotional intelligence, and community engagement across various educational modalities.

The existing literature has discussed the positive and negative roles of Gen-AI in education for both teachers and students. For instance, for teachers, the positive outcomes include efficient grading (Nazaretsky et al., 2022), curriculum mapping (Raskob et al., 2025), time saving (Rensfeldt & Rahm, 2023), and reduced efforts (Alwaqdani, 2025). The negative consequences for teachers include biases in the training data (Gauthier et al., 2022), a lack of intellectual ideas (Celik et al., 2022), and other issues that lead to unfair or discriminatory outcomes (Bit et al., 2024; Harry, 2023; Omughelli et al., 2024). For students, the positive outcomes are problem-solving (Einarsson et al., 2024), personalized learning experiences (Chan, 2023), enhancing creativity (Najmiddinova, 2024), etc. The negative outcomes related to students are a lack of ethical considerations (Saeidnia, 2023), reduced human interaction (Crawford et al., 2024), a lack of intellectual diversity (Batubara et al., 2024; Habiba et al., 2025; Najmiddinova, 2024), etc. Previous studies have also shed light on how Gen-AI in education and academia enhances social development among students and teachers by fostering collaboration, communication skills, and self-esteem through adaptive, game-like activities (Grace et al., 2023; Z. Xu, 2024). Previous studies often neglect the crucial divergence in perceptions between teachers and students regarding Gen-AI’s influence on social development across both online and physical learning environments. In particular, it is not yet known how teachers and students utilize Gen-AI in their teaching and learning methods to enhance their social development (Alwaqdani, 2025; Şenel, 2024; R. Zhang et al., 2025). Therefore, this work of research answers the following questions:

1. How do teachers perceive the role of Gen-AI integration into specific teaching methods in enhancing their social development, and how do these perceptions compare between the contexts of online classes and physical classrooms?
2. How do students perceive the role of Gen-AI integration into specific learning methods in enhancing their social development, and how do these perceptions compare between the contexts of online classes and physical classrooms?
3. What are the key commonalities and divergences in the perceptions of teachers and students regarding Gen-AI’s influence on social development across online learning environments versus physical classroom settings?

The literature has suggested a total of nine teaching methods that are used by teachers in both online and physical classes. These methods are Communicative Language Teaching (CLT), the Direct Method, Task-Based Language Teaching (TBLT), Content and Language Integrated Learning (CLIL), Community Language Learning (CLL), the Audiolingual Method, the Grammar-Translation Method, Suggestopedia, and the Silent Way. For this study, only five methods—namely, CLT, TBLT, DM, CLIL, and CLL—are encompassed, given their relevance to our study. These methods collectively best define social developments within today’s educational learning environment. Similarly, several teaching methods are being reported for understanding the learning methods of students, but our study utilizes Communicative Learning (CL), Immersive Learning (IL), Task-Based Collaborative Learning (TBCL), Content Integrated Learning (CIL), and Community-Based Reflective Learning (CBRL). The literature has substantially investigated AI’s role in educational contexts; yet, its role in shaping teachers and students’ perceptions across modalities still needs to be determined. Thus, we aim to fill this gap by providing a broader analytical framework by investigating and comparing the perceptions of both groups toward Gen-AI at various levels. In exploring this complex interplay with contrasting perceptions, our findings provide critical insights.

Our study provides several significant outcomes. First, it provides a comprehensive analysis of teachers’ and students’ perceptions regarding the benefits of Generative AI both individually and collectively. This provides a deep insight into how Generative AI can be integrated into educational models that can significantly contribute to social development. This approach of advancing from general assumptions to an empirical analysis is fundamental for designing pedagogical strategies and policy recommendations. Aligning and implementing such strategies with existing learning models can result in enhanced social competencies, which are relevant to the needs of teachers and students. Moreover, by capturing the perceptions of both teachers and students simultaneously, our study enriches the literature with a more guided structure that can be used to develop advanced AI-centric educational models. These models can enhance social confidence, learning, and communication between teachers and students.

Empirically, our study expands the existing knowledge on Generative AI by examining its transformative role in shaping the perceptions of teachers and students. It highlights the usefulness of AI in terms of the social development of the learning community, such as the contribution of AI in community building, social confidence, communication, and emotional intelligence. Moreover, investigating the perceptions of both teachers and students in a single model provides a comparative analysis, which, in the previous literature, has received limited attention. Additionally, investigating the role of AI in both traditional and online classes contributes to a cross-modal understanding of AI’s educational impact. These findings are equally important for practitioners because, by elucidating the commonalities and discrepancies in these perceptions, our research provides actionable insights for educators, policymakers, and curriculum developers. With these findings, effective educational models can be designed that integrate Gen-AI in a way that can foster social growth and academic achievements.

## 2. Literature Review

The integration of online and physical classes transforms how teachers and students interact, especially as they increasingly use Gen-AI in education (Huang et al., 2023; Lacka et al., 2021; Son et al., 2025).

### 2.1. Teaching Methods: Teachers’ Perspectives

Teaching methodologies emphasize social development. The methodologies, which include CLT, the DL, TBLT, CLIL, CLL, the Audiolingual Method, the Grammar-Translation Method, Suggestopedia, and the Silent Way, help to improve social development and are being adapted through Gen-AI-driven tools in online and physical educational settings (Belda-Medina, 2021; Ji et al., 2023). Teachers follow different methodologies, depending on their classes and potential advantages and perceived social improvement (Ma, 2021).

The Grammar-Translation Method is one of the earliest methods that focuses on translating texts and memorizing vocabulary and grammar rules. It began in the 1700s and 1800s and was initially used to teach classical languages like Latin and Greek before being utilized to teach modern languages (Bovée, 1919; Dakhalan & Tanucan, 2024). The primary focus of this method is on reading and writing, while less emphasis is placed on speaking and listening skills (Boswell, 1972). As a reaction against the Grammar-Translation Method, the DL emerged during the late 18th and 19th centuries (Boswell, 1972; Bureković et al., 2023). It advocates for directly teaching the target language, rather than using the native language (Boswell, 1972). Emphasis is given to oral interaction, visual aids, and inductive grammar teaching (Dakhalan & Tanucan, 2024).

The Audiolingual Method, which is based on behaviorist psychology and structural linguistics, gained popularity in the mid-20th century (1940s–1950s) (Boswell, 1972). It emphasizes repetitive drills and pattern practice to develop correct pronunciation (Castagnaro, 2006). Grammatical accuracy can also be developed through this method (Dakhalan & Tanucan, 2024). Boswell draws attention to this method in 1972: audio materials alongside mimicry represent key focusing elements for this method. The audiolingual method had certain problems that led to the rise of CLT in the 1970s (Savignon, 1991; Yousaf et al., 2017).

The CLT method employs real-life language, focusing on fluency and the ability to convey messages in an effective manner, rather than perfection. Grammatical role-playing and discussions, coupled with problem-solving tasks, represent common activities in CLT classrooms (Savignon, 1991; Yousaf et al., 2017).

Furthermore, in the 1980s, TBLT emerged as a new approach. It builds on CLT ideas by organizing lessons so that students may complete their work (Bureković et al., 2023; González-Lloret, 2017). TBLT seeks to integrate language skills within a meaningful context as it promotes accuracy along with fluency, and the learning processes of teachers (González-Lloret, 2017).

CLIL, which was developed in the 1990s, combines language learning with studying other subjects (Morton, 2016). Within CLIL, students use the target language to learn a subject like science or history. This method seeks to increase subject knowledge and linguistic skills all at once (Jaleniauskiene, 2021).

Around the same period, Charles Curran developed CLL in the 1970s, and it is based upon the principles of creating a supportive and collaborative educational setting. The method views the teacher as being a counselor and the learners as being collaborators, and this fosters a sense of community (La Forge, 1971).

Similarly, Suggestopedia, developed by Georgi Lozanov, was created back in the 1970s. It optimizes the process of learning by way of relaxation as well as suggestion. A positive and stress-free classroom atmosphere with drama, art, and music incorporated within it is created (Waliyuddin et al., 2024). The aim is to tap into learners’ untapped mental reserves, which makes learning more efficient, along with being an enjoyable experience. Finally, the Silent Way method was introduced by Caleb Gattegno during the 1970s. Learner autonomy as well as discovery are indeed relied upon by it. Because of the fact that the teacher uses some gestures, some visual aids, and Cuisenaire rods, they remain relatively silent to guide students. Their own rules and understanding are developed by learners as they actively explore the language (Sasi et al., 2020).

However, with the emergence of technologies and, especially, AI, some methods have gained more popularity. For instance, CLT stresses real-life communication and interactive teaching and learning (Savignon, 1991). With the integration of Gen-AI in online and physical classes, it impacts teachers’ social development, offering adaptability, collaboration competence, cultural competence, emotional intelligence, and ethical decision-making (Placklé et al., 2022). Moreover, CLT fosters community building and social confidence (Alharbi, 2024; Placklé et al., 2022).

The DM stresses engaging in communication in the target language, which occurs without translation, and it focuses on oral skills as well as inductive learning (Dakhalan & Tanucan, 2024). Teachers may improve social confidence as well as cross-cultural communication skills with students through AI-integrated tools like speech recognition, plus AI-generated visual aids within online and physical classes (Lacka et al., 2021). Teachers develop cognitive flexibility because they adjust lessons in real time to learner responses. Gen-AI also reduces repetitive tasks, which supports their well-being and balances work and social life in online and physical teaching environments (Xia et al., 2024).

On the other hand, TBLT stresses meaning-focused, real-world tasks that engage learners in collaborative language use (Guangwei, 2003; Willis & Willis, 2013). When TBLT is initiated with Gen-AI in online and physical classes, teachers refine collaboration competence as well as problem-solving adaptability and ethical decision-making by designing and moderating diverse learner groups (Ye et al., 2025). Teachers also gain social confidence and emotional intelligence when they carefully monitor group dynamics that Gen-AI feedback tools improve (Tran, 2025).

Furthermore, the CLIL integrates language learning with subject-based instruction, promoting interdisciplinary engagement in both online and physical classes (Navés, 2009). With AI-supported content delivery, such as adaptive materials and virtual cultural simulations, teachers enhance cognitive flexibility, cultural competence, and community-building by tailoring lessons to diverse learner needs (Jäppinen, 2005). Managing AI-enhanced interdisciplinary projects in online and physical environments strengthens their collaboration competence and enables cross-generational social skills through richer classroom interactions (Coyle, 2007).

Lastly, CLL, grounded in counseling theory, positions teachers so that they may support students in the role of counselors in classes that are both online and physical. This creates a trust between teachers and students leading to enhanced collaboration between both (Larsen-Freeman, 1986; Wassell et al., 2013). Improved collaboration between teachers and students results in enhanced critical thinking skills, whereas, if this collaboration is accompanied by AI-based emotional analytics such as sentiment-detection and peer-feedback platforms, it results in a significant enhancement in teachers’ emotional stability, community building, and ethical decision-making based on real-time insights (W. Xu, 2022). In fact, with the help of these tools, teachers are equipped with capabilities that strengthen their self-confidence and well-being in online and physical classrooms (Boswell, 1972).

Building on these traditional and modern methodologies, recent research explores how teachers adapt these methods because Gen-AI improves online and physical classrooms (Bureković et al., 2023; Dakhalan & Tanucan, 2024). From the teacher’s perspective, when AI is integrated with selected methods such as the CLT, the DM, the TBLT, CLIL, and CLL, it influences social development. Teachers not only adjust their pedagogical approaches but also refine their social development factors that include adaptability, emotional intelligence, collaboration, and cultural competence. All these methods are important, considering the class environments and facilities. However, in the edge of AI, particular methods such as CLT, DL, TBLT, CLIL, and CLL have become significantly important for social development. The emergence of AI has shaped teaching methods, which have positively influenced social development. For instance, using AI technologies in CLT helps teachers in building communication competencies (Adem & Berkessa, 2022). DL enables their social functions in society and, therefore, positively contributes to the self-enhancement of the teachers and community (Öngören, 2022). TBLT improves the interaction of teachers with students (both in online and physical classes), and, therefore, it significantly improves ongoing professional development (C. Chen, 2023). Similarly, CLIL also plays a key role in the integration of teachers and students in particular contents and discussion, which, in turn, positively affects self-enhancement (Hussain, 2022). CLL enables teachers to engage in practical discussion and debate about the community to understand the real picture, and, thus, it spurs overall social development (W. Xu, 2022). These methods gained popularity with the development of technology; yet, the research on the integration of AI tools in both online and physical classes of these methods is lacking. Hence, our research contributes to the literature and shapes policy implications for better social development practices.

### 2.2. Learning Methods: Students’ Perspectives

Similar to teachers, students also have distinct perceptions and use different learning techniques (Sánchez-Ruiz et al., 2023). The integration of Gen-AI into online and physical classes has transformed not only the way teachers practice but also the way students socially develop and interact (Conklin & Hartman, 2014). Gen-AI changes social confidence as well as well-being, verbal with non-verbal communication, and community engagement when Gen-AI mediates physical and online educational settings. Gen-AI has redefined many traditional and modern methods for CL, IL, TBCL, CIL, and CBRL, fostering enhanced social, cultural, and cognitive competencies (Borg, 2003). CL is grounded in interaction-focused methodologies like CLT and the DM, stressing real-life language use, fluency, and direct engagement in the target language (Dakhalan & Tanucan, 2024; Savignon, 1991).

When Gen-AI integrates into these methods, students see a reduction in social anxiety along with an increase in social confidence because Gen-AI chatbots, speech recognition, and real-time feedback systems let them practice conversations in low-pressure opportunities (Beketov et al., 2024; L. Liu et al., 2022); Gen-AI-assisted role-playing and interactive simulations are highlighted in CLT. These are tools that help learners to feel more capable within group interactions (Abedin et al., 2022). Gen-AI strengthens cultural literacy and multilingual communication skills since students improve verbal and nonverbal communication through real-time corrections of pronunciation and body language from visual aids (Brandizzi, 2023). Students benefit from digital tool utilization because Gen-AI-generated personalized prompts, along with pronunciation models, encourage self-directed speaking practice (El Shazly, 2021).

Furthermore, Engaging Learning, paralleling both Suggestopedia and the Audiolingual Method, betters language acquisition using sensory-rich and experiential activities (Boswell, 1972; Waliyuddin et al., 2024). Gen-AI-powered virtual reality and augmented reality tools allow students to engage in simulated real-world environments. These tools also increase cultural awareness with educational literacy while improving community engagement (Havrda, 2020). Engaging Gen-AI environments encourages emotional well-being since they make language learning enjoyable and stress-free, echoing Lozanov’s original premise of reducing anxiety (Waliyuddin et al., 2024).

Moreover, studies indicate AI-based platforms with engaging storytelling and gamification promote multilingual learning (Ma, 2021; Sánchez-Ruiz et al., 2023). Students switch languages easily and build cognitive flexibility, plus improved social confidence in multicultural environments (Festman, 2021).

TBCL, based on TBLT, highlights real-world authentic tasks and problem-solving (Willis & Willis, 2013). Collaboration competence as well as community engagement are improved by the integration of Gen-AI into TBLT (C. Chen, 2023). Gen-AI tools like group-matching algorithms, along with real-time feedback systems, result in more balanced participation within group work (Bygate, 2016). Students develop critical discussion and problem-solving skills when working in diverse groups, as Gen-AI monitors the interactions and suggests equitable turn-taking strategies (Fan & Zhong, 2022). AI-supported TBLT fosters social confidence along with verbal/nonverbal communication. Students’ interaction with Gen-AI instantly provides feedback, which encourages reflection and improves digital literacy for collaborative tools (Anwar et al., 2020; J. Zhang & Zhang, 2024). Emotional well-being also improves because AI reduces group-work anxiety by providing scaffolding and adaptive task difficulty (Khine, 2024).

Moreover, CLIL principles merge subject knowledge and language learning (Hu et al., 2023). AI-enhanced CLIL environments through adaptive content delivery, intelligent tutoring systems, and virtual cultural simulations expose students to interdisciplinary, multilingual contexts, improving their educational and cultural literacy (Sercu, 2025). Therefore, educationally and digitally literate students are able to develop skills that are pivotal for critical discussions and multilingual competencies, whereas, in the presence of Gen-AI, these skills are further enhanced in the form of subject-specific vocabulary and cultural nuances in real time, empowering students with an opportunity for cross-cultural and cross-generational communication (Morton, 2024). Ultimately, it results in strengthened community engagement with the help of AI-supported CLIL projects that involve combined problem-solving tasks on global issues, improving ethical awareness and social confidence (Lo, 2019). Therefore, the CBRL model, inspired by CLL, is derived from the principles of trust, collaboration, and reflective dialogue (Nguyen et al., 2024).

Numerous studies have linked Gen-AI tools—such as peer-feedback platforms, sentiment analysis, and others—with trust, empathy, and social confidence among students (Bauer et al., 2023; Gelaidan & Abdullateef, 2017; Yilmaz & Yilmaz, 2023). With the help of Gen-AI, learners are able to track emotional climates in group activities that promote their overall well-being and reduce their social anxiety (Beketov et al., 2024; Lund et al., 2024). CLL, when integrated with AI, leads to community engagement and digital citizenship, which empowers students with capabilities that are essential for participation in online reflective journals, cultivating their cultural literacy and social responsibility (Havrda, 2020).

Overall, AI tools have significantly improved the popularity of these particular methods (CL, IL, TBCL, CIL, and CBRL) among students due to their key role in shaping social development. For instance, when integrated with AI tools, CL mitigates the ambiguity among students and enhances their social communication (Silva et al., 2021). IL benefits students in social and emotional development by engaging them in real tasks (Tan et al., 2022). TBCL encourages students and enhances their collaborative skills in society and integrates them in groups (Aksoy-Pekacar, 2024). CIL assists the content development skills of students, and, thus, results in ongoing improvement in their learning skills (Cimermanová, 2021). CBRL brings positive social change among students and enables their interaction with real community issues and practices. This integration enriches their social development and social skills (Morrison, 2021). While previous studies have contributed to the literature, the importance of these learning methods in students’ social development on the edge of AI in physical and online classes has been overlooked. Hence, our research enriches this aspect to articulate the policy implications in a better way.

In summary, traditional and modern teaching methods, along with CLT, TBLT, CLIL, and CLL, and student-centered approaches like CL, IL, TBCL, CIL, and CBRL, have greatly shaped social development in education because researchers have integrated Gen-AI, and they increasingly adapt these methods in online and physical settings to improve collaboration, cultural competence, social confidence, and emotional well-being among teachers and students. However, a limited amount of research explores in a systematic manner how Gen-AI transforms these teaching as well as learning methods so as to foster social development, presenting a critical gap that this study addresses.

## 3. Methodology

### 3.1. Research Design

This research aims to understand the importance of AI in education and compare the distinct perceptions of teachers and students regarding AI’s role in their social development aspects in both online and physical classroom settings. We wanted to know about the unique outcomes and perceptions, and to evaluate whether AI is more beneficial for social development in physical or online classes for teachers and students in particular teaching methods. To achieve these objectives, this research’s thematic analysis employs an inductive approach to understanding the patterns and behaviors of teachers in improving their social development by employing Gen-AI in teaching methods. Similarly, we wanted to understand how students, as a result, enhance their social development through these methods (classes) by using Gen-AI. Considering our research objectives, an inductive approach utilizing thematic analysis would be highly suitable, allowing for the identification of recurring patterns in how academicians (teachers and students) utilize Gen-AI to enhance social development through various teaching methods in both online and physical classes. The primary aim of this research is to understand how various teaching methods in online classes and physical classes contribute to the social development of teachers and students through Gen-AI usage.

This study adopts an inductive qualitative research approach. An inductive approach is chosen because the goal was to derive theoretical insights and patterns directly from the rich, descriptive data provided by the participants (teachers and students) (Braun & Clarke, 2019; Guba & Lincoln, 1994). This allows for the emergence of themes that are grounded in the lived experiences and expressed opinions of individuals. The data for this study are drawn exclusively from teachers’ and students’ views on the role of AI in teaching methods to transform social development. These insights and thoughts offer direct insights into personal experiences, observations, and perspectives. The questions are provided in Appendix A.

We used purposive sampling to obtain the most relevant and important insights. For instance, we wanted to collect data from teachers and students who were part of both online and physical classes and were involved in these particular teaching and learning methods. We focused on teachers and students in China, as there are both online and physical classes in various universities. China has been significantly focused on AI development; for instance, the AI tool DeepSeek V3.2-Exp is one of the examples that shows the Chinese’s focus and improvement in AI. We first ensured that the respondents engaged in the teaching and learning methods in the relevant online and physical classes. We hired four enumerators for the data collection who already knew the interviews and research but were also trained for particular topics to enhance validity. A sample interview was conducted with each enumerator, making it clear for further data collection. With the help of the enumerators, we reached out to 20 teachers (10 from online classes and 10 from physical classes), and 40 students (20 from online and 20 from physical classes). The sample size is deemed to be appropriate based on previous studies where 40 students (Y. Liu et al., 2022) and 20 teachers (Kim, 2024) were interviewed. To gain specified information and avoid overlapping, we interviewed those teachers for online classes who were actively engaged in online teaching as compared to physical classes. Similarly, for physical classes, we selected those teachers who exclusively engaged in teaching physical classes. The same methodology was followed for students. The participating students and teachers were from the departments of natural science, computer science, and social science, making the study robust and reliable. Students’ ages range from 18 to 30 years (as they were enrolled in universities), while teachers’ ages range from 30 to 53 years in the sample. It demonstrates that older people (students) do not significantly focus on AI in their teaching methods.

Prior to executing complete interviews, the reliability and validity of the interview questions were confirmed by a pilot test. To address ethical issues such as voluntary involvement and data protection, we acquired informed permission forms to facilitate the collection of participant opinions via interviews. TurboScribe Transcription Software 2024 was used for the transcription to guarantee rapid and accurate audio data conversion. The software NVivo 14 was used for coding and analyzing the resultant transcripts. This method discerned themes and patterns within the data.

We informed the respondents about the purpose of the research and assured them that the data would be exclusively used for research purposes, rather than for public or commercial purposes. We also informed the participants that the interviews would be recorded for research purposes, which could be later used for data analysis. Therefore, we recorded all the interviews in audio form, which were later transcribed for coding and analysis. Based on the recording, the average time for the interview was 55 to 69 min.

Thematic analyses were employed to systematically identify, analyze, and report patterns (themes) within the qualitative data. This method is chosen for its flexibility and its ability to capture the complexity of meaning within a dataset. The steps followed for this analysis involved following steps:

### 3.2. Organization of Research

- **Familiarization with the Data:** All interviews provided were read twice to capture deep understanding of their context and content. Carefully engaging at the beginning of the process is termed crucial for early impressions and potential areas of interest.
- **Initial Coding:** Each reflection was systematically analyzed and further segmented into meaningful units. Each part was coded with a concise description, reflecting its context. For instance, teaching methods used in both online and physical classrooms were categorized into AI-specific social development statements. Similar approach was adopted for students in both online and physical classrooms.
- **Searching for Themes:** After coding, each part was further reviewed and grouped into broader distinct themes based on patterns of meaning across the dataset. These connections were based on commonalities between each group’s codes.
- **Reviewing Themes:** In the next step, each potential theme was critically analyzed against the original coded extracts as well as with the entire dataset. This step ensured the accuracy of data representation. Data was refined, merged, or split where necessary. Doing so ensured coherence and distinctiveness between each set of data. Inter-code reliability of themes and codes was specifically emphasized by dividing interviews into two groups, each evaluated by two authors. Upon cross-checking, the data resulted in high correlation. Cohen’s Kappa or percentage agreement quantitative agreement metrics were used where necessary. This ensured the preservation of uniqueness and relatedness of the themes.
- **Defining and Naming Themes:** After finalization of themes, each theme was individually defined and given descriptive name that comprehensively explained the meaning and “story” it encapsulated.
- **Producing the Report:** Finally, the findings were presented in a coherent manner, supported by direct quotes from reflections of themes, reflecting the context in which they were stated.

## 4. Results and Findings

The transcriptions were evaluated in two ways. First, we extracted sample statements related to the integration of Gen-AI and social development in online and physical classes for teachers and students. In this section, we ensured that social development could be enhanced using AI in the classes (online and physical), irrespective of using any particular teaching method. Second, we performed comparative analyses between teachers and students to evaluate the role of AI, focusing on the particular teaching and learning methods for each social development.

### 4.1. AI and Social Development (Teachers)

Drawing on the transcriptions, we derived sample statements demonstrating how a teacher’s social development can be fostered using Gen-AI, specifically differentiated for online and physical class settings, and linked to various teaching methodologies:

#### 4.1.1. Adaptability and Cultural Competence

Online Classrooms: “By using AI to analyze and adapt diverse online cultural content for my teaching methods and lessons, I’ve significantly enhanced my own cultural competence, becoming much more adaptable to the varied backgrounds of my global online students”.

Physical Classrooms: “Integrating AI tools to generate culturally relevant scenarios and task-based activities, my physical classroom has directly improved my adaptability in addressing the nuanced cultural needs present among my physical students”.

#### 4.1.2. Emotional Intelligence

Online Classrooms: “Utilizing AI to analyze real-time engagement data from online lessons helps me to discern the subtle emotional states of students remotely, thereby deepening my own emotional intelligence in responding empathetically to their virtual learning experiences”.

Physical Classrooms: “When AI tools provide immediate feedback on student comprehension during a physical session, it allows me to better gauge classroom atmosphere and adjust my approach, leading to improved self-regulation and enhanced emotional intelligence in real-time interactions”.

#### 4.1.3. Social Confidence

Online Classrooms: “Automating routine tasks with AI in my online class has freed up time, allowing me to engage in more personalized virtual interactions with students, which has notably boosted my social confidence in my role as an online educator”.

Physical Classrooms: “Mastering new AI presentation tools for my lessons in the physical classroom has given me a stronger sense of professional efficacy, translating into greater social confidence when I present new concepts or strategies to students and colleagues face-to-face”.

#### 4.1.4. Cognitive Flexibility

Online Classrooms: “AI’s ability to generate diverse instructional materials and pedagogical suggestions for online classes and sessions has pushed me to think beyond traditional virtual approaches. This constant exposure to new ideas has profoundly improved my own cognitive flexibility as an online teacher”.

Physical Classrooms: “When AI offers multiple solutions to a teaching challenge during a physical lesson based on real-time classroom observations, it forces me to consider different perspectives and adapt my instructional design, which enhances my cognitive flexibility in problem-solving on the fly”.

#### 4.1.5. Well-Being and Work-Social Life

Online Classrooms: “By automating repetitive online tasks like drafting rubrics or providing initial feedback using AI, my workload for online meetings and classes has significantly reduced, directly contributing to my personal well-being and a healthier balance in my work–social life outside of screen time”.

Physical Classrooms: “The efficiency gained through AI handling administrative tasks for my physical classes has allowed me more time to engage in professional development, pursue personal interests, and spend more quality time with family and friends, positively impacting my overall well-being and work–social life by reducing in-school administrative burden”.

#### 4.1.6. Digital Tools Utilization

Online Classrooms: “My daily reliance on AI for lesson planning, content creation, and virtual classroom management methods (like online CLM and DM) has made me exceptionally proficient in utilizing a wide array of advanced digital tools, which is essential for my professional growth in the online educational landscape”.

Physical Classrooms: “I’m now not just using basic digital tools; I’m actively integrating AI-powered smart boards and interactive platforms for physical classrooms (e.g., TBLT and CLIL), showcasing a deeper mastery of digital tool utilization and innovating my physical classroom environment”.

#### 4.1.7. Community Building

Online Classrooms: “Leveraging AI-powered virtual communication platforms for our online group work has made it easier for me to connect with online learners and parents, thereby helping me build a stronger sense of community in a remote educational setting”.

Physical Classrooms: “Using AI-facilitated collaborative tools to manage shared projects in my classes has strengthened my professional connections with colleagues and contributed to my sense of belonging within our school’s physical teaching community”.

#### 4.1.8. Ethical and Inclusive Decision-Making

Online Classrooms: “As I incorporate AI into curriculum design for online courses, I’m constantly prompted to consider algorithmic biases and data privacy concerns for a global audience. This process has inherently sharpened my own ethical and inclusive decision-making regarding online content and student data”.

Physical Classrooms: “Using AI to generate diverse ethical dilemmas for discussion in my physical classes has made me more conscious of fostering inclusive decision-making practices, ensuring all voices are heard and respected in the physical classroom environment”.

#### 4.1.9. Cross-Generational Social Skills

Online Classrooms: “Being at the forefront of AI integration in online teaching often means I bridge communication gaps between tech-savvy students and less familiar parents or administrators, thereby enhancing my cross-generational social skills in explaining new online educational technologies”.

Physical Classrooms: “Demonstrating and explaining AI tools used directly in the physical classroom for physical sessions to diverse stakeholders—from younger students to older colleagues—has required me to adapt my communication style, which has significantly improved my cross-generational social skills within the school community”.

#### 4.1.10. Collaboration Competence

Online Classrooms: “Collaborating with other educators globally on AI-integrated curriculum projects for online task-based learning has fostered a much stronger sense of virtual teamwork and significantly boosted my collaboration competence in a technologically evolving remote environment”.

Physical Classrooms: “Using AI as a shared resource in team-teaching scenarios for physical sessions, especially for generating differentiated materials, has refined my ability to work effectively with others, improving my collaboration competence in designing and delivering innovative lessons within the physical school setting”.

### 4.2. AI in Teaching Methods for Social Development Categories (Teachers)

**Figure 1:**
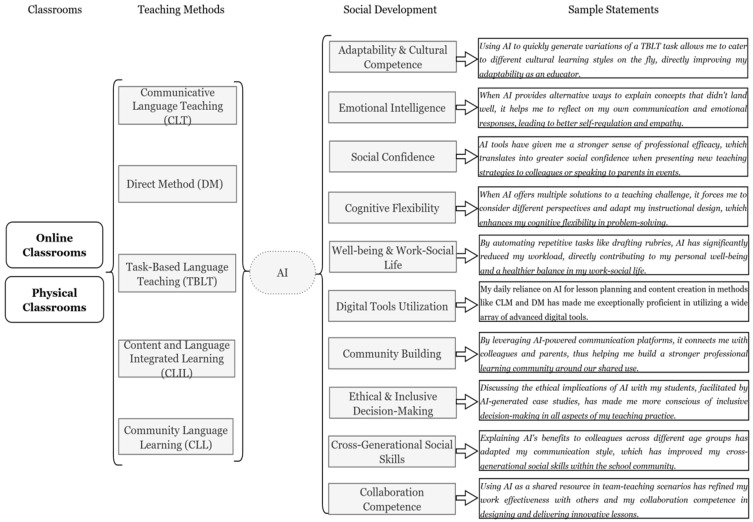
Teachers’ perceptions in online and physical classes.

**Figure 2:**
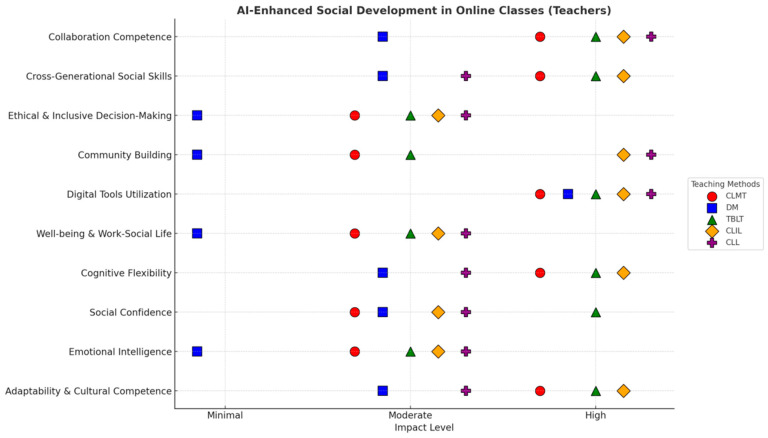
Teaching methods and social development in online classes (teachers).

**Figure 3:**
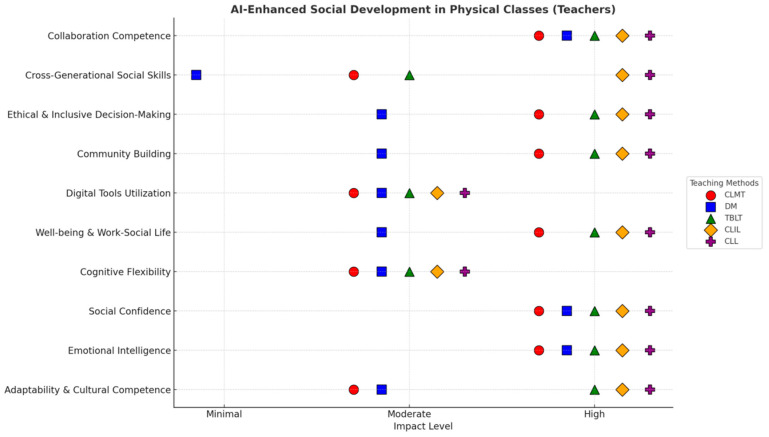
Teaching methods and social development in physical classes (teachers).

Figure 1 illustrates how various social development aspects can be improved when teachers integrate Gen-AI into different learning methods in both online and physical classroom environments. Figure 2 and Figure 3 assess the level of development (High, Moderate, or Minimal) for ten specific social development aspects across five different learning methodologies: CLT, DM, TBLT, CLIL, and CLL.

#### 4.2.1. Social Development Across Teaching Methods in Online Classroom (Teachers)

Figure 2 reflects teachers’ perceptions of how using Gen-AI within online learning methods contributes to students’ social development. It demonstrates that Gen-AI-enhanced online learning is seen as highly effective in fostering several key social skills:

*Highly Developed Aspects:* Emotional intelligence, social confidence, well-being and work–social life, community building, ethical and inclusive decision-making, and collaboration competence are frequently rated as “high” across multiple learning methods in online classes. This suggests that Gen-AI, when used in these contexts (e.g., through communicative learning tools or task-based collaborations), significantly enhances students’ abilities to understand and manage emotions, interact confidently, maintain balance, connect with peers, make responsible decisions, and work effectively in teams.

*Moderately Developed Aspects: *Figure 2 highlights that adaptability and cultural competence, cognitive flexibility, digital tools utilization, and cross-generational social skills generally show a “moderate” level of development. While still beneficial, these areas might require more targeted AI applications or specific pedagogical approaches to reach a “high” level in an online setting.

*Minimally Developed Aspects:* Only cross-generational social skills show a “minimal” development under the “DM”. This implies that certain online Gen-AI applications might not be as effective in bridging generational communication gaps.

#### 4.2.2. Social Development Across Teaching Methods in Physical Classroom (Teachers)

Figure 3 highlights the perspectives of teachers on AI-integrated learning methods and their impact on social development within traditional, in-person teaching settings. The results indicate that the levels of perceptions differ from those of online environments, demonstrating the unique strengths and challenges:

*Highly Developed Aspects*: Figure 3 illustrates that adaptability and cultural competence, digital tool utilization, cognitive flexibility, collaboration competence, and cross-generational social skills are frequently rated as “High.” This demonstrates that the use of AI in physical classrooms results in essential outcomes for students. For example, such students can easily adjust to new environments, adapt digital tools, think flexibly, adjust to each age group, and collaborate with them. In particular, community building showed a high development in “CLL” method.

*Moderately Developed Aspects*: In our investigation, several social development aspects were scored as “moderate” (see, Figure 3). These aspects included social confidence, emotional intelligence, individuals’ well-being, social–work life, ethical and inclusive decision-making, and, under certain methods, community building. This indicates the context-driven nature of AI in physical classroom environments. It is arguable that the AI support in traditional classrooms might be moderate as compared to online settings.

*Minimally Developed Aspects*: Amongst all aspects of development, emotional intelligence, community building, work–social life and well-being, and ethical and inclusive decision-making showed ‘‘minimal’’ development under the “DM” method. In line with the online settings, this highlights the limitations of certain AI integration methods, suggesting that, in different settings, AI fosters social development differently.

Overall, both Figure 2 and Figure 3 illustrate the distinct nature of Gen-AI at various levels in different learning methods. While teachers perceive AI as a prominent tool for enhancing social development, their view differs in specific settings, ranging from AI having a high to minimal impact in online and physical classes.

### 4.3. AI and Social Development (Students)

Based on the student sources and interviews, which established the types of classes, learning methods, the role of Gen-AI, and the 10 categories of social development, here are sample statements from a student’s perspective demonstrating how they use AI within different learning methods to enhance their social development:

#### 4.3.1. Reducing Social Anxiety

Online Classrooms: “When participating in online classes, AI tools help me structure my thoughts and even practice my responses beforehand, which has significantly reduced my social anxiety about speaking up in virtual group discussions.”

Physical Classrooms: “Using AI to simulate various social scenarios in learning methods has allowed me to practice conversations in a safe space, making me feel much less anxious when I have to speak in front of my classmates, thereby reducing my social anxiety.”

#### 4.3.2. Critical Discussion

Online Classrooms: “In our online sessions, AI-powered prompts always challenge my initial ideas and encourage me to consider different viewpoints, which has greatly improved my ability for critical discussion on complex topics.”

Physical Classrooms: “AI helps us access a wide range of analytical frameworks for our learning and experience, pushing me to analyze information more deeply and engage in more thoughtful and critical discussions with my peers in class.”

#### 4.3.3. Social Confidence

Online Classrooms: “Practicing presentations with AI feedback on my delivery in learning methods environments has made me feel much more prepared and articulate, boosting my social confidence when I have to speak during online video calls.”

Physical Classrooms: “Thanks to AI guiding me through collaborative tasks in learning methods, such as CIL, I feel more knowledgeable and capable when working with classmates face-to-face, which has genuinely increased my social confidence in group settings.”

#### 4.3.4. Verbal and Non-Verbal Communication

Online Classrooms: “The AI in my online session and works provides real-time suggestions on my tone and clarity, directly enhancing my verbal communication skills during remote interactions.”

Physical Classrooms: “Through AI-powered role-playing scenarios in classes and workshops, I’ve learned to pay more attention to subtle cues like body language, which has improved my understanding and use of verbal and non-verbal communication in the classroom.”

#### 4.3.5. Well-Being

Online Classrooms: “AI personalizes my learning pace and identifies areas where I need extra support in online methods (such as IM), which reduces my stress and contributes significantly to my overall well-being as an online learner.”

Physical Classrooms: “When AI handles some of the repetitive organizational tasks for our classes and course assignments, it frees up my time and reduces my workload, positively impacting my well-being and making learning feel more manageable.”

#### 4.3.6. Digital Tools Utilization

Online Classrooms: “Engaging in online learning methods with AI-powered collaboration platforms has made me highly proficient in using various advanced digital tools for teamwork and content creation.”

Physical Classrooms: “The physical seminars and classes often use AI-enhanced interactive displays and learning platforms in the classroom, constantly developing my skills in digital tools utilization far beyond basic computer use.”

#### 4.3.7. Opportunities

Online Classrooms: “AI has opened up incredible opportunities by connecting me with peers from different countries for online classes and seminars, allowing me to participate in virtual exchanges and learn about diverse cultures firsthand.”

Physical Classrooms: “Through AI, our projects can now analyze real-world data and connect us with experts outside the classroom, creating amazing opportunities for practical learning and community engagement.”

#### 4.3.8. Multilingual Learning

Online Classrooms: “Using AI for real-time translation and pronunciation practice in online learning has been transformative; I can now comfortably engage with materials and discussions in languages other than my native one, significantly advancing my multilingual learning.”

Physical Classrooms: “AI-powered language apps integrated into our classes allow us to practice with AI tutors and even communicate with native speakers, greatly enhancing my multilingual learning capabilities within the classroom setting.”

#### 4.3.9. Literacy (Educational and Cultural)

Online Classrooms: “AI helps me break down complex academic texts and interpret cultural nuances in my online learning, and in particular CL assignments, which has dramatically increased my educational and cultural literacy.”

Physical Classrooms: “When AI provides instant context and background for the diverse historical and cultural topics we cover in a physical class; it deepens my understanding and significantly enhances my cultural literacy.”

#### 4.3.10. Community Engagement

Online Classrooms: “The AI-moderated discussion forums in our online learning, such as TBCL, have made it easier and more rewarding to contribute my ideas and support my classmates, boosting my sense of community engagement in our virtual classroom.”

Physical Classrooms: “AI helps us identify local needs for our classes, such as in the CBRL projects, which encourages me to get more involved in outreach and directly strengthens my community engagement beyond the school walls.”

### 4.4. AI in Teaching Methods for Social Development Categories (Students)

**Figure 4:**
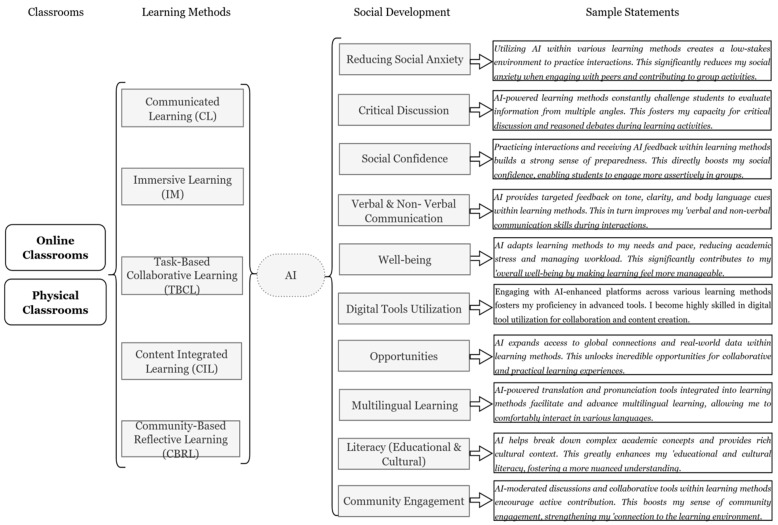
Students’ perceptions in online and physical classes.

**Figure 5:**
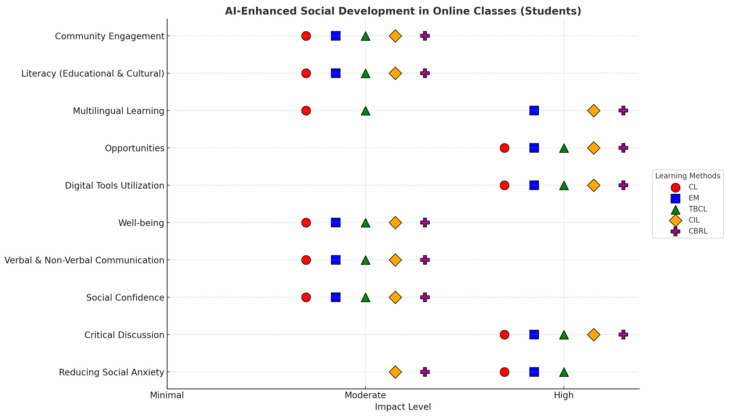
Learning methods and social development in online classes (students).

**Figure 6:**
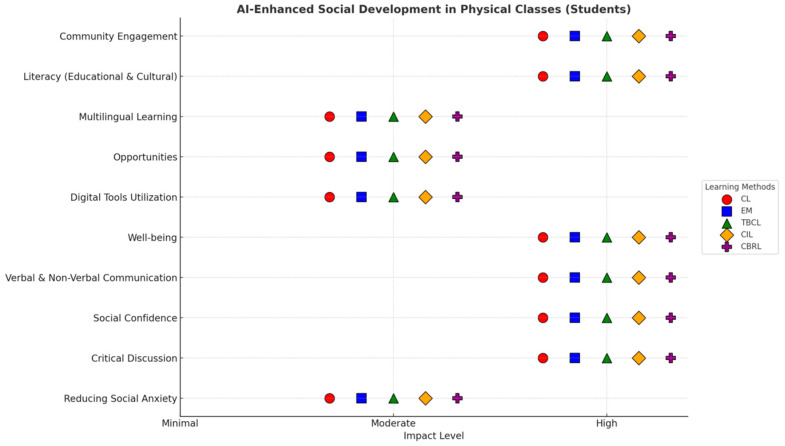
Learning methods and social development in physical classes (students).

Figure 4 reflects the views of students on AI-integrated learning methods. From the figure, it is observed that students perceive several methods of learning to be more effective when AI models accompany them. They perceive AI as a supporting tool in their social development in both online and physical classroom settings. However, in contrast to the teachers’ perception, the development perception ranged from High to Moderate across different learning methodologies (see Figure 5 and Figure 6).

#### 4.4.1. Social Development Across Learning Methods in Online Class (Students)

Figure 5 and Figure 6 indicate how students perceive AI as a critical tool for improving their social development in different learning methods. It is pertinent to note that online AI engagement particularly fosters certain skills, while providing moderate benefits for others (see Figure 6).

*Highly Developed Aspects:* Critical Discussion is the highest reported method of all, suggesting that students perceive AI as a significant contributor to their critical discussion by improving their deep analytical and rational debate skills. This impact is observed across all learning methods (CL, IM, TBCL, CIL, and CBRL). Consistent with expectations, Digital Tool Utilization is also reported as “high” across all methods, suggesting that online AI engagement requires the mastery of digital tools and interfaces, which, ultimately, boost the students’ proficiency in each aspect. Similarly, access to new opportunities in all online models is rated “High” by students. This substantiates the key role of AI in expanding the access to resources that are fundamental to the educational experience. One of the key findings is that of social anxiety, which is rated “High” under the CL, IM, and TBCL methods. It can be deduced that, in the presence of AI, the students perceive learning environments as less intimidating, which supports their confidence-building, particularly in activities that involve online communication channels. Gen-AI can provide a platform for facilitating language acquisition and practice in these contexts.

*Moderately Developed Aspects:* Across all online learning models, verbal and non-verbal communication, social confidence, well-being, community engagement, and literacy (cultural and educational) are rated as moderate. This suggests that, although students view AI’s influence as substantially enhancing, they do not perceive its role in these development dimensions to be highly effective. Thus, AI might be able to support these aspects, but, to transform it into a highly perceived learning method, human interaction or specific pedagogical interventions are necessary.

#### 4.4.2. Social Development Across Learning Methods in Physical Class (Students)

Figure 5 provides insights into how students perceive Gen-AI’s contribution to their social development within a traditional, physical classroom setting. Here, the strengths of AI integration shift, highlighting different areas of impact compared to the online environment.

*Highly Developed Aspects:* Consistent to an online class environment, the results indicate that the critical discussion across all physical classroom methods is perceived as highly relevant. This finding reinforces the importance of Gen-AI in the development of critical thinking, regardless of the type of learning environment. Moreover, the self-confidence construct demonstrated similar reports across all methods, implying that the use of Gen-AI in traditional face-to-face learning provides students with opportunities to have live feedback that directly spurs their confidence in social interaction. Similarly, both the communications—verbal and non-verbal—are highly rated in physical classroom settings. This suggests that the integration of Gen-AI enhances the communication skills of students, through which they are capable of conveying their message effectively and are able to obtain feedback through real-time interactions. Students also rate well-being ‘’High” across all methods in physical classroom methods. It could be explained through the dynamic capabilities of AI which empowers students to personalize their learning, reducing their academic stress by providing timely support. Such support is critical for their overall learning experience in a physical classroom environment. Both Cultural and Educational Literacy are perceived as “High” across all methods. It indicates the potential of AI in providing a diverse perspective, richer content, and adaptive learning paths. With access to such information, students are able to understand the deep insights of various cultures and subjects in physical classrooms. Interestingly, community engagement is also consistently rated as “High” across all methods in physical classrooms, illustrating the attributes of AI tools in the facilitation of collaborative projects, a shared learning experience, and group discussions, that leads to a strengthened community among students.

*Moderately Developed Aspects:* Amongst moderately developed aspects, digital tool utilization, multilingual learning, social anxiety, and opportunities were identified across all physical classroom methods. This finding diverges from the observations of the online class environment, where opportunities and digital tool utilization were rated as “High.” This converse finding can be attributed to the nature of physical classes where students see more emphasis on social interaction. Thus, students might perceive AI as essential for reducing their anxiety or creating new digital/multilingual avenues, but its impact can be perceived as less pronounced.

Collectively, these findings demonstrate the positive role of AI in social development, where different settings underscore its various functionalities. From the findings, it can be inferred that, in digital learning environments, Gen-AI catalyzes the process of knowledge gathering and digital mastery, aiding students in opportunity hunting and enhancing their critical thinking skills. Further, Gen-AI mitigates the impact of social stress through providing a safe space in certain communication contexts. Moreover, AI plays a constructive role in boosting confidence through improved social cohesion. This ultimately nurtures their social confidence, well-being, communication skills, and capabilities to engage within their learning community. In essence, the perspectives of both teachers and students emphasize the significant, albeit context-dependent, role of Gen-AI in fostering holistic social development.

### 4.5. Comparison Between Teachers and Students

In summary, there are several similarities and differences observed in the perspectives of both teachers and students regarding the influence of Gen-AI on social development. These similarities and differences are consistently reported in both online and physical classes.

#### 4.5.1. Similarities in Social Development Indicators

*Critical Discussion:* Both teachers and students consistently perceive AI as having a high impact on critical discussion across various learning methods in both online and physical classroom environments (see Figure 3, Figure 4, Figure 5 and Figure 6). This suggests a strong consensus that Gen-AI facilitates analytical thinking and debate.

*Digital Tools Utilization (Online)*: From Figure 3 and Figure 5, it is evident that both teachers and students share a collective understanding of Gen-AI usage for social development while emphasizing digital utilization in online learning. This positive influence of Gen-AI is justified because the learning environments inherently rely on digital proficiency.

*Social Confidence & Well-being (Physical)*: Both social confidence and well-being are perceived as contributing factors in social development by teachers and students. This induces the supportive role of AI in providing nurturing environments that enhance students’ assurance and overall welfare.

*Community Building/Engagement (Physical):* Higher community building or engagement was perceived in the presence of AI by both the groups within physical classes. This implies that AI improves the collaboration and communication skills of teachers and students in physical classes.

#### 4.5.2. Differences in Social Development Indicators

*Broader “Soft Skills” (Teachers’ Emphasis)*: Our findings report several divergences in the comparative analysis of both groups. For instance, in teachers, emotional intelligence, ethical and inclusive decision-making, and competence are highly influenced by Gen-AI in both physical and online learning environments. However, in groups of students, similar aspects—including social confidence, well-being, and community engagement—were rated moderate in online classes. This underscores that AI, in the teachers’ group, has a more profound impact on their interpersonal and ethical dimensions than it has on the students’ groups.

In physical classes, teachers also highlight adaptability and cultural competence, cognitive flexibility, and cross-generational social skills as areas of high development, which are not explicitly listed or as consistently highly rated by students in their information.

*Practical and Foundational Skills (Students’ Emphasis):* Students uniquely emphasize opportunities and reducing social anxiety as highly developed in online classes through Gen-AI. These aspects are not explicitly categorized or consistently rated as high by teachers in the online environment. This suggests students experience direct benefits in terms of access and comfort when AI is integrated online.

In physical classes, students perceive AI as resulting in highly developed verbal and non-verbal communication and literacy (educational and cultural), which are not directly mirrored as highly developed social aspects in the teachers’ figure. This highlights the students’ focus on AI’s practical support for communication and knowledge acquisition.

*Digital Tools Utilization (Physical Classroom Discrepancy):* Interestingly, while both agree on the high digital tool utilization online, teachers perceive digital tool utilization as “high” across all methods in physical classrooms, whereas students consistently rate it as “moderate” in the same setting. This could suggest that, while teachers actively integrate Gen-AI tools, students might feel their existing digital literacy is sufficient, or they do not perceive the physical classroom as the primary driver for *new* digital skill development when AI is present.

*“Minimal” Development (Teachers’ Sole Observation):* Only the teachers’ perceptions include “minimal” development for certain social aspects, particularly under the DM in both online and physical settings. For instance, teachers see “cross-generational social skills” as minimally developed with DM online, and several core social skills (emotional intelligence, well-being, community building, ethical and inclusive decision-making) as minimal with DM in physical classes. Students do not report any “minimal” development for any social aspect in their figures. This implies that teachers are more discerning about the limitations of AI in fostering certain social skills under specific pedagogical approaches.

In essence, while both teachers and students agree on AI’s strong role in fostering critical thinking and digital proficiency (especially online), teachers tend to perceive AI as a powerful tool for developing a broader spectrum of “soft skills” like emotional intelligence, ethical decision-making, and collaboration. Conversely, students often highlight AI’s direct, tangible benefits, such as creating new opportunities, reducing anxiety, and improving foundational communication and literacy skills. It is like teachers see Gen-AI as refining the underlying engine of social interaction, while students see it as enhancing the practical features of the vehicle.

## 5. Discussion

This research explores how teachers and students utilize Gen-AI in their teaching (CLT, DM, TBLT, CLIL, and CLL) and learning methods (CL, IL, TBCL, CIL, and CBRL) for social development in both online and physical classes. Unlike previous studies that have focused on the direct relationship between Gen-AI and social development (e.g., P. Chen et al., 2024; Qian et al., 2024), as well as AI and learning performance (e.g., Ezzaim et al., 2024; Wu & Yu, 2024), this research integrates the role of AI in various teaching and learning methods for social development among teachers and students in both online and physical classrooms. Our findings provide heterogeneous as well as homogeneous insights in terms of social developments, and, therefore, enrich the literature on AI, teaching and learning methods, and social development indicators.

We found ten social development aspects among teachers, namely, adaptability and cultural competencies, emotional intelligence, social confidence, cognitive flexibility, well-being and work–social life, digital tool utilization, community building, ethical and inclusive decision making, cross-generational social skills, and collaboration competencies, and ten among students such as reducing social anxiety, critical discussion, social confidence, verbal and non-verbal communication, well-being, digital tools utilization, opportunities, multilingual learning, literacy, and community engagement.

Previous studies have emphasized only narrow aspects of social development among teachers (e.g., Al-Zahrani & Alasmari, 2024; Lai et al., 2023; Sharples, 2023) and students (e.g., Huang, 2021; Kim et al., 2022). Moreover, previous studies have either focused on only teachers (e.g., Borg, 2003; Chiu et al., 2023; Yousaf et al., 2017) or students (e.g., Anani et al., 2025; Chan & Hu, 2023; Huang, 2021). We integrated both teachers and students into our model to enrich the literature and articulate the results in a better way.

We found that teachers and students use Gen-AI in their teaching methods to enhance social development aspects from various perspectives. These findings match with those in previous studies (Aguilar-Esteva et al., 2023; Beketov et al., 2024; Khan et al., 2021; Kim et al., 2022; Salas-Pilco et al., 2022) that using AI in teaching and learning methods by teachers and students enhances and adds to their social development in various perspectives. However, previous studies have emphasized the direct relationship and association of Gen-AI in educational outcomes and social development. We used AI as an integrated approach to understand its importance in teaching methods and learning methods for social development.

Some studies show that using AI in teaching and learning methods could negatively affect creativity and critical thinking (Lin & Chen, 2024; W. Liu & Wang, 2024; Nazaretsky et al., 2022). It creates and leads to anxiety among students due to a lack of trust (Lin & Chen, 2024). It can negatively influence human interaction in a teaching environment, thus leading to a lack of trust (Alwaqdani, 2025). The negative consequences create doubts among teachers and attenuate their confidence, thus adversely influencing students’ performance and social development. Therefore, policymakers and educational specialists need to create balancing strategies for AI use in education. They need to think about the dual aspects of AI tools to reduce their negative role in social development.

### 5.1. Implications for Practice

Based on the insights derived from the perceptions of both teachers and students, here are some implications for its use in practice.

#### 5.1.1. Practical Implications for Teachers

*In Online Class Settings:* Teachers should design learning activities that explicitly leverage AI tools for critical discussion and analysis, as both teachers and students consistently perceive Gen-AI to have a high impact in this area, for example, using AI-powered tools for argument mapping or debate preparation. Although teachers perceived AI to be highly beneficial for their emotional intelligence, ethical and inclusive decision making, and collaboration competencies, students view its significance as moderate in this regard. This suggests that teachers can actively use AI-supported scenarios like AI-simulated dilemmas, and collaborative project management tools that foster their soft skills. By doing so, teachers can understand and elevate students’ perception that can result in actual development. While students already perceive digital tool utilization to be important in online settings, teachers must ensure that Gen-AI is integrated in a way that it enhances the digital literacy of students, which can improve their problem solving skills. Moreover, teachers must use AI for facilitating group projects through which their social interaction skills can be improved.

*In Physical Class Settings:* Teachers should integrate AI to actively enhance students’ social confidence, verbal and non-verbal communication, well-being, and community engagement. This could involve Gen-AI for feedback on presentations, role-playing scenarios, or collaborative project management. Students find Gen-AI to be highly beneficial for cultural and educational literacy. Hence, Gen-AI can potentially be used to enhance the existing pool of knowledge that provides diverse perspectives enriched with critical information. Moreover, higher community engagement is a pivotal aspect within the students’ group. Thus, the enriched pool of information can easily be disseminated within groups that can result in productive outcomes. Therefore, Gen-AI has the potential to facilitate collaboration, and shared learning experiences, which can further lead to group cohesion.

Meanwhile, from the teachers’ perspective, Gen-AI can be used for the development of students’ cultural competence, adaptability, cross-generational social skills, and cognitive flexibility. Such developments are fundamental for their creative problem solving by borrowing support from exposure and diverse viewpoints. It is pertinent to note that teachers themselves perceive few methods (such as DM) to be slightly influential because of their limited role in shaping their critical social development aspects, like well-being, emotional intelligence, and ethical and inclusive decision-making. Thus, it is suggested that we should avoid the over-reliance on aforementioned aspects. In fact, such AI models should be used by them such that it aids them in overcoming these weaknesses. Doing so can result in constructive approaches that foster their skills. The results indicate a moderate rating of digital tool utilization in contrast to the teachers’ group, who rated it “High.” Therefore, teachers must ensure that the AI tools utilized within classrooms are advanced. In this way, it will improve their digital skills and literacy, which, in the long run, will improve their skill growth in learning environments.

Our findings underscore some concerning trends. For instance, AI tools with regard to social development play a damaging role in both physical and online classes. This can decrease the overall effectiveness of AI usage in learning environments. Therefore, the teacher needs to thoroughly investigate the paradoxical tensions of AI usage. Moreover, they must emphasize the transparency and ethics of AI usage in learning environments. Using AI with such precautions can positively contribute to the social development of students and the community.

#### 5.1.2. Practical Implications for Students

*In Online Class Settings:* To achieve academic goals, students must understand the mechanisms through which their academic performance can be enhanced with the use of Gen-AI. Recent developments in AI have provided students with essential tools that are crucial for their academic activities. Students can use AI to promote critical discussions because of its potential to generate ideas that support deeper analytical thinking. Students need to explore AI to expand their pool of learning resources. With access to exclusive resources, they can enhance their unique educational experiences. For mitigating the impact of social anxiety over educational performance, students should use communicative models that are driven by Gen-AI. Such models have a higher tendency to reduce anxieties that are experienced in learning environments. In addition, students’ development and multilingual practices can be leveraged by advanced AI tools (like IM, CBL, and CLIL). AI’s impact on social confidence, well-being, community engagement, and verbal and non-verbal communication is perceived as moderate. Therefore, students must emphasize direct interaction with teachers in order to fully develop these crucial social dimensions.

*In Physical Class Settings:* In physical classrooms, AI has enormous potential to enhance students’ social confidence, well-being, verbal and non-verbal communication skills, and literacy skills. Therefore, using relevant AI models can significantly affect their overall social development. Furthermore, students must seek opportunities to engage in discussions that are supported by AI tools (like using chatbots, language translators, and others) to improve their community engagement. Students intending to use AI for multilingual learning, opportunities, digital tool utilization, and reducing social anxiety must realize that AI alone is not enough to address these issues. They need some supplementary resources along with AI to ensure maximum social development.

Briefly, implementing AI in educational learning is a nuanced approach. While teachers view AI as an essential tool for complex social skills and learning outcomes, students emphasize its practical benefits, such as its ease of access and usage, comfort, and core skill refinement. It indicates that both teachers and students perceive AI from different dimensions. The role of teachers and students can be understood by viewing the teacher as an architect and the student as a builder, working on the same construction site. Teachers develop strategies, design the framework, and plan activities that are essential for the development of their knowledge, social skills, and emotional growth. Students, on the other hand, consistently engage with AI to practice skills, complete tasks, and apply what they learn. Therefore, students use AI to reinforce and implement the teachers’ plan. Therefore, for both teachers and students, using AI improves the overall learning environment.

Some previous studies highlight the negative impact of AI on critical thinking which can contribute to the students’ anxiety. This underscores the importance of the moral and ethical usage of AI tools. Students must analyze the consequences of overlooking the ethical boundaries of educational learning in the context of AI usage. Knowing the consequences of AI, students can mitigate its negative influence over their academic performance and social development.

### 5.2. Contributions to the Study

Our study significantly contributes to the existing body of research by providing a more comprehensive and comparative analysis of teachers’ and students’ perspective regarding Gen-AI. This provides a subtle and nuanced perspective of Gen-AI usage in the learning environment, which presents a multifaceted impact of AI on the social development of both teachers and students that, in the previous literature, is widely neglected.

From the teachers’ point of view, the role of AI is highly context driven, suggesting that the impact of AI can vary in different learning environments. For instance, teachers perceive AI to be highly supportive for their emotional intelligence in online classes; however, its impact fades in physical classrooms. In a similar vein, their perception of AI’s influence over their community building is high in online settings; yet, it drops to a moderate rating in physical interactions. On the other hand, teachers associate a high impact of AI with digital tool utilization in physical classrooms, but only a moderate impact in online settings. This distinct perspective on how Gen-AI enhances teachers’ social development across modalities offers crucial insights into their evolving roles and needs.

From the student perspective, this research meticulously highlights the commonalities and critical discrepancies in perceptions between teachers and students regarding AI’s contribution to student social development in both online and physical classrooms. For example, in online classes, while teachers perceive a high impact of AI on social confidence and community building, students rate these same aspects as only moderate. Conversely, in physical classes, students perceive a high impact on social confidence and community engagement, whereas teachers perceive Gen-AI’s impact on social confidence and community building as being only moderate. These divergent views across stakeholders and modalities emphasize that the actual benefits of AI for social development are experienced and interpreted differently, underscoring a critical gap in prior research.

By elucidating these commonalities and discrepancies, this research moves beyond generalized assumptions to offer actionable insights for educators, curriculum developers, and policymakers. The findings facilitate the creation of targeted pedagogical strategies and policy recommendations that effectively leverage AI to cultivate essential social competencies in a manner that genuinely resonates with the experiences and needs of both teachers and students. This granular understanding is akin to having a detailed map that shows not just the roads, but also the different perspectives of drivers and passengers, allowing for a more efficient and satisfying journey for everyone involved in the educational landscape.

Despite these contributions, this work of research has a few limitations that could be addressed in future studies. First, this research focused on Chinese teachers and students; future research should extend the model to other countries. We only focused on interviews; future studies can gather more diverse and mixed data to evaluate the model. Our model demonstrates the positive influence of AI in social development; future researchers should go in-depth and evaluate the negative consequences of AI tools on social development. It will articulate the findings and implications for better practices.

### 5.3. Conclusions

Based on interviews of 20 teachers and 40 students from both online and physical classes, our results indicate significant differences in perceptions regarding AI’s impact on social development, both between teachers and students, and across online versus physical learning environments.

In online classes, teachers consistently perceive a high impact of Gen-AI on emotional intelligence, social confidence, community building, ethical and inclusive decision-making, and collaboration competence. However, their perception of AI’s impact on digital tool utilization is only moderate in online classrooms. In physical classes, teachers perceive a high impact of AI on adaptability and cultural competence, cognitive flexibility, digital tool utilization, cross-generational social skills, and collaboration competence. Strikingly, teachers perceive Gen-AI’s impact on emotional intelligence as only moderate and community building as only moderate in physical classes, compared to their high perception in online classes for these same aspects.

In online classes, students consistently perceive a high impact of AI on digital tool utilization, opportunities, critical discussion, and reducing social anxiety. However, their perception of AI’s impact on social confidence, verbal and non-verbal communication, well-being, literacy, and community engagement remains moderate in online settings. Conversely, in physical classes, students perceive a high impact of Gen-AI on social confidence, verbal and non-verbal communication, well-being, critical discussion, literacy, and community engagement. Notably, students perceive AI’s impact on digital tool utilization as only moderate in physical classes, a stark contrast to their high perception in online settings.

A primary finding is the unaddressed complexity arising from the distinct perceptions of both teachers and students. For instance, students perceive AI to have a high impact on digital tool utilization in online classes, but this perception drops to moderate in physical classes, while teachers perceive the opposite trend, rating digital tool utilization as moderate in online settings, yet high in physical classrooms. Teachers perceive a high impact of AI on emotional intelligence and collaboration competence in online classes, yet rate emotional intelligence as only moderate and collaboration competence as moderate in physical classes. Furthermore, while teachers perceive Gen-AI to have a high impact on social confidence and community building in online settings, students rate these aspects as moderate in the same context. Conversely, in physical classes, students perceive AI to have a high impact on social confidence, verbal and non-verbal communication, and well-being, areas where teachers’ perceptions are often moderate or even minimal for certain methods.

In summary, the findings underscore that the perceived role of AI in fostering social development is not uniform. Instead, it is highly dependent on the stakeholder (teacher vs. student) and the learning environment (online vs. physical). These divergences highlight that the effectiveness and specific benefits of AI integration for social growth are experienced and interpreted very differently by key participants in the educational process, akin to two different people watching the same play but focusing on different characters and themes.

## Data Availability

The data presented in this study are available upon request from the corresponding author. The data are not publicly available due to privacy restrictions.

## Appendix A

*General Introductory Questions (For Both Teachers and Students)*

Can you please describe which teaching methods/learning methods you use and why? Examples.

Can you describe your current experience using Generative AI (Gen-AI) tools in your teaching/learning activities within your online classes?

Can you describe your current experience using Gen-AI tools in your teaching/learning activities within your physical (in-person)/online classes?

Which specific AI tools do you most frequently utilize in your educational setting, and how often do you use them? And why?

How does a typical online class session in which you integrate AI, compared to a typical physical class session, affect your outcomes (e.g., social development)?

*The questions below were asked specifically regarding the integration of Gen-AI into one or more of the five focused teaching methods (CLT, DM, TBLT, CLIL, and CLL) and differentiated by class modality (online vs. physical).*

Suggested Interview Questions
How does the use of AI in adapting lessons (e.g., culturally relevant content) for physical classrooms (or online classes) specifically influence your personal adaptability as an educator?
When utilizing AI tools to analyze real-time student engagement or sentiment in an online setting, how does this feedback affect you?
By mastering new AI presentation or content generation tools for a physical classroom, how has this technical proficiency translated into greater professional skills when interacting with students or colleagues?
Describe a situation in a physical lesson where AI offered multiple, competing pedagogical suggestions. How did this challenge force you to enhance your cognitive flexibility or ability to adapt your instruction on the fly?
How do AI-involved tasks for online meetings or in-person meetings specifically contribute to your personal well-being and in your work–social life?
How has your daily reliance on Gen-AI for content creation and virtual classroom management in online/physical teaching methods increased your proficiency in advanced digital tool utilization?
How does leveraging AI-powered tools in your physical classes/online classes strengthen your professional connections and sense of belonging within the community?
When incorporating AI into the curriculum design for online courses/physical classes for an audience, how do you deal with biases, privacy, and your ethical decision-making?
Describe how Gen-AI tools in the physical classroom/online classes diversify or enhance your social skills.
How have AI-integrated curriculum projects for online/physical task-based activities specifically fostered your sense of virtual teamwork and boosted your collaboration competence?

Here are some questions for students in five learning methods: CL, IL, TBCL, CIL, and CBRL. The questions explore the student’s direct, personal perception of AI’s impact on their social skills and experiences.

*The questions below were asked specifically regarding the integration of Gen-AI into one or more of the five focused learning methods (CL, IL, TBCL, CIL, and CBRL) and differentiated by class modality (online vs. physical).*

Suggested Interview Questions
When participating in online/physical group discussions, how do Gen-AI tools (like chatbots or real-time practice systems) help you structure your thoughts or practice responses? Is it helpful in a positive way or negative way, and how?
In your online sessions (or physical), how do AI-powered prompts or analysis frameworks challenge your initial ideas and build your confidence?
How has practicing presentations or collaborative tasks with AI feedback in a physical/online classroom setting made you feel more prepared and articulate?
Describe how the AI in your online/physical sessions or workshops provides suggestions on your tone, clarity, or body language. How does this real-time feedback enhance your communication skills?
How does Gen-AI’s ability to personalize your learning pace or handle your assignments in online/physical classes impact your overall academic and social well-being?
How has engaging with AI-powered collaboration platforms within your online/physical methods made you more proficient in utilizing various advanced digital tools for teamwork and content creation? Please provide an example.
How has AI opened up new opportunities for you, such as connecting you with peers from different countries for online classes or allowing you to analyze real-world data in physical projects?
Describe how using AI has helped you comfortably engage with materials or discussions in languages other than your native one in physical classes (or online). Does it affect your social enhancement and how?
How does AI help in complex academic texts or provide instant context for diverse historical and cultural topics in your physical/online class, thereby enhancing your knowledge and literacy?
How have AI-moderated discussion forums or AI-supported social skills and development in your physical classes (or online learning) made it easier for you to contribute your ideas and strengthen your sense of community engagement?
